# Effects of Astraflavonoid A and Astraside C from the Aerial Part of *Astragalus membranaceus* on TNF-α-Induced Human Dermal Fibroblasts

**DOI:** 10.3390/plants14091358

**Published:** 2025-04-30

**Authors:** So-Ri Son, Kang Sub Kim, Mingoo Jun, Dae Sik Jang, Sullim Lee

**Affiliations:** 1 College of Pharmacy, Kyung Hee University, 26 Kyungheedae-ro, Dongdaemun-gu, Seoul 02453, Republic of Korea; allosori@khu.ac.kr; 2 College of Korean Medicine, Gachon University, 1342 Seongnamdae-ro, Sujeong-gu, Seongnam 13120, Republic of Korea; kang-sub@gachon.ac.kr; 3 Department of Biomedical and Pharmaceutical Sciences, Graduate School, Kyung Hee University, 26 Kyungheedae-ro, Dongdaemun-gu, Seoul 02453, Republic of Korea; mingoojun0104@khu.ac.kr; 4 Department of Life Science, College of Bio-Nano Technology, Gachon University, 1342 Seongnamdae-ro, Sujeong-gu, Seongnam 13120, Republic of Korea

**Keywords:** *Astragalus membranaceus*, flavonol glycosides, anti-skin-aging, antioxidant, anti-inflammation

## Abstract

The present study investigates the anti-skin-aging properties and bioactive compounds of the aerial parts of *Astragalus membranaceus*, which are typically discarded as agricultural waste. Liquid chromatography–mass spectrometry analysis identified flavonoid glycosides as the major constituents of the aerial parts of *A*. *membranaceus* extract. Two principal flavonoids, astraflavonoid A (**1**) and astraside C (**2**), were isolated using repetitive chromatography. Compounds **1** and **2** demonstrated antioxidative properties, reducing reactive oxygen species and matrix metalloproteinase-1 levels in human dermal fibroblasts upon stimulation with TNF-α. Specifically, astraside C (**2**) inhibited the expression of pro-inflammatory cytokines interleukin-6 and interleukin-8, whereas astraflavonoid A (**1**) did not affect their expression. Additionally, the expression of inflammatory mediators such as nuclear factor kappa B and cyclooxygenase-2 (COX-2) was increased by **1**, whereas it was suppressed by **2**. Furthermore, in silico molecular docking experiments confirmed that compound **2** effectively binds to COX-2. These findings suggest that the aerial parts of *A*. *membranaceus* contain bioactive flavonol glycosides with promising anti-skin-aging properties, offering valuable use as agricultural byproducts.

## 1. Introduction

Recent developments in food and nutrition science have led to a global increase in life expectancy, which has reached 80 years within a century [1,2]. As life expectancy has increased, the importance of a healthy lifespan has also been recognized. Consequently, there is a growing interest in maintaining physical health and appearance, with an emphasis on preventing skin-aging symptoms, such as wrinkles and xerosis.

Tumor necrosis factor-α (TNF-α) is a key biomolecule in the process of skin aging [3]. Environmental factors, such as ultraviolet (UV) radiation, trigger TNF-α, which is closely related to aging and inflammatory skin diseases [4]. In dermal fibroblasts, TNF-α regulates the production of reactive oxygen species (ROS). The overproduction of ROS can activate signaling pathways such as mitogen-activated protein kinase (MAPK)/nuclear factor kappa B (NF-κB) and activated protein-1 (AP-1) in response to oxidative stress [5]. This produces collagenase and matrix metalloproteinase-1 (MMP-1), which decompose collagen fibers and become the main cause of wrinkle formation. Among various matrix metalloproteinases, MMP-1 (collagenase-1) plays a direct role in the degradation of type I and III collagens, which constitute the primary structural framework of the dermal extracellular matrix. In contrast, MMP-3 (stromelysin-1) exhibits broad substrate specificity, targeting proteoglycans, laminin, and fibronectin, and can activate other MMPs, including proMMP-1 and proMMP-9. MMP-1 was selected as the primary marker in this study given its direct involvement in collagen breakdown and wrinkle formation. Additionally, TNF-α stimulates the release of other proinflammatory cytokines such as interleukin-6 (IL-6) and interleukin-8 (IL-8), contributing to inflammatory skin disease.

Some investigations have revealed the potential of plants and their bioactive compounds to have anti-skin-aging properties by regulating oxidative stress and inflammatory processes, particularly those stimulated by TNF-α. For example, studies on *Cosmos caudatus* and its phenolic compounds have demonstrated anti-skin-aging effects through the inhibition of MMP-1 and MMP-3 activities triggered by TNF-α [6]. Similarly, brazilin from *Caesalpinia sappan* exhibited anti-inflammatory properties in human keratinocytes (HaCaT) treated with TNF-α [7]. However, most research to date has focused on UV-induced skin aging and the TNF-α-induced skin-aging process, and its inhibitors are relatively underexplored [6]. Therefore, a comprehensive investigation of various medicinal plants and their phytochemicals against TNF-α is needed to identify more specific targets for anti-skin-aging interventions.

*Astragalus membranaceus* Bunge (family: Fabaceae) is a medicinal plant native to northern China and is cultivated throughout Northeast Aisa [8]. Its roots (Astragali radix) have been widely used as dietary supplements and ingredients in health-promoting foods and beverages owing to their antioxidant, anti-inflammatory, and immunostimulatory properties [9]. Currently, only the roots of *A*. *membranaceus* are utilized for nutritional applications. However, considering that the aerial parts of *A*. *membranaceus* typically reach heights of 40–70 cm, a significant amount of these aerial parts is discarded as agricultural waste [10,11]. To address this issue, a recent study has focused on repurposing the aerial parts of *A*. *membranaceus* as value-added products to employ the entire plant more effectively. Pharmacological research has shown that the aerial part of *A*. *membranaceus* exhibits antioxidant, antibacterial, and anti-leukopenia activities in in vitro and in vivo experiments [10,11,12]. In addition, bioactive compounds have also been isolated from the aerial parts of *A*. *membranaceus*. The flavonol glycosides, astraflavonoid A and B, enhance glucose consumption in L6 myotubes [13]. Furthermore, astrooleansaponins, first isolated from the aerial part of *A*. *membranaceus*, reduced triglyceride levels in sodium oleate-treated HepG2 cells [14]. Despite this scientific evidence, the anti-aging effects of the aerial parts of *A*. *membranaceus* have not been explored. In our preliminary studies, 30% ethanol extract from the aerial parts of *A*. *membranaceus* inhibited ROS production and reduced MMP-1 expression. Therefore, the present study focused on the potential of these aerial parts as a source of bioactive compounds with anti-skin-aging properties. Initially, to profile the characteristic compounds in the aerial part of *A*. *membranaceus* extract, UHPLC-LTQ-MS analysis was conducted, followed by the isolation and elucidation of major compounds by MS analysis. To validate the biological activities of the aerial parts of *A. membranaceus*, the isolated compounds were assessed for their antioxidant and anti-inflammatory effects using TNF-α-induced normal human dermal fibroblasts (NHDFs). This model is a well-established in vitro system that mimics the inflammatory and oxidative stress-related mechanisms involved in skin aging. TNF-α-stimulated NHDFs are widely used to evaluate the anti-aging potential of candidate compounds, particularly those that target ROS production, MMP-1 secretion, and pro-inflammatory cytokines.

## 2. Results and Discussion

### 2.1. Effects of A. membranaceus Extract on NHDF

UV radiation activates TNF-α receptors on the surface of skin cells and promotes the release of inflammatory cytokines, causing skin tissue damage [3]. TNF-α overproduces ROS and activates MAPK and NF-κB in NHDF. This increases MMP-1 expression, decomposes collagen, and induces an inflammatory response [15,16,17]. The ECM of the skin, which mainly consists of fibronectin, collagen, and elastin fibers, is degraded by aging-related proteins, such as MMPs [18]. MMPs decompose ECM components, causing skin relaxation and a reduction in interstitial fluid, whereas MMP-1 decomposes type 1 collagen and thins the dermis, causing a loss of elasticity and accelerating the formation of wrinkles [19,20]. Therefore, the suppression of ROS and MMP-1 secretion is an important factor in preventing skin aging.

**Figure 1:**
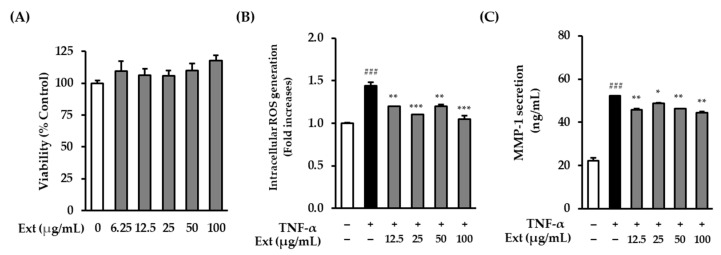
Effects of *A. membranaceus* extract on NHDF viability (**A**), ROS production (**B**), and MMP-1 secretion (**C**). (**A**) NHDFs were treated with various concentrations of the extract for 24 h and cell viability was measured using the EZ-Cytox assay. (**B**) NHDFs were pretreated with the extract for 24 h and then stimulated with TNF-α (20 ng/mL) for 15 min, and ROS production was measured immediately thereafter. (**C**) NHDFs were pretreated with the extract for 24 h and then stimulated with TNF-α (20 ng/mL) for 12 h. MMP-1 secretion levels were quantified using ELISA. Data are presented as mean ± SEM (*n* = 3). ^###^ *p* < 0.001—untreated group versus TNF-α-induced group. * *p* < 0.05, ** *p* < 0.01, and *** *p* < 0.001—sample treatment group versus TNF-α-induced group.

Before analyzing the effects of the *A. membranaceus* extract on NHDFs, the cell viability of NHDFs was investigated. It was confirmed that the extract was not toxic at concentrations of 100 μg/mL or less (Figure 1A). ROS formation in NHDFs induced by TNF-α was then investigated to determine whether the extract of *A. membranaceus* exhibited antioxidant efficacy. The ROS levels in the TNF-α treatment group increased significantly by 1.44 ± 0.04-fold (*p* < 0.001) compared with the control group, whereas the addition of *A. membranaceus* extract (100 μg/mL) to the TNF-α-treated NHDF culture reduced the amount by which ROS levels increased to 1.05 ± 0.04-fold (*p* < 0.001) (Figure 1B). Interestingly, ROS levels showed a slight increase at 50 μg/mL compared to those at 25 μg/mL. This phenomenon may be attributed to nonspecific cellular stress induced by intermediate concentrations of the extract, as has been observed in other phytochemical studies. However, this increase was not statistically significant, and a significant reduction in ROS generation was observed at 100 μg/mL, indicating a dose-dependent antioxidant effect.

Furthermore, to determine whether *A. membranaceus* extract reduced the secretion of collagen-degrading enzyme MMP-1, the amount of MMP-1 secretion in NHDFs induced by TNF-α was investigated. The TNF-α treatment group showed a significant increase of 52.28 ± 0.09 ng/mL (*p* < 0.001) compared to the control group, whereas the inclusion of *A. membranaceus* extract (100 μg/mL) reduced the amount of MMP-1 that was secreted to 44.55 ± 0.48 ng/mL (*p* < 0.01) (Figure 1C). However, the MMP-1 inhibitory effect of the extract appeared to plateau at higher concentrations. This may reflect the saturation of the bioactive constituents of the extract at the receptor level or within intracellular signaling pathways. Similar concentration-dependent effects have been observed in other studies on natural products.

### 2.2. Identification of Compounds from the Aerial Parts of A. membranaceus

**Figure 2:**
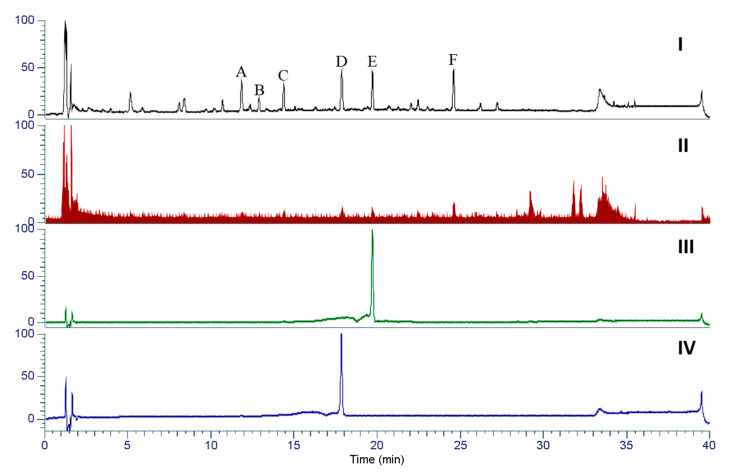
UHPLC-PDA-MS chromatograms of the 30% EtOH extract from the aerial parts of *A*. *membranaceus* (ASME-A) and compounds **1** and **2**. I: PDA chromatogram of ASME-A. II: Base peak chromatogram of ASME-A. III: PDA chromatogram of astraflavonoid A isolated from ASME-A (*t*_R_ 19.72 min, compound **1**). IV: PDA chromatogram of astraside C isolated from ASME-A (*t*_R_ 17.85 min, compound **2**).

To identify the bioactive principles, we analyzed the 30% EtOH extract of the aerial parts of *A*. *membranaceus* (ASME-A) using UHPLC-PDA-MS. This led to the detection of six major compounds, labeled peaks A–F (Figure 2). By comparing the retention times and the MS^2^ spectra of these peaks with the reported data in the literature, SpectraBase (https://spectrabase.com, accessed on 20 July 2024), and our in-house database, we identified the following compounds: quercetin-3-*O*-glucoside (A, *t*_R_ 11.85 min), kaempferol-3-*O*-rutinoside (B, *t*_R_ 12.35 min), kaempferol-3-*O*-glucoside (C, *t*_R_ 14.38 min), and rhamnocitrin-3-*O*-glucoside (F, *t*_R_ 24.58 min) (Table 1). Peaks D and E were tentatively identified as a flavonol glycoside, but their exact identification remained unclear based on the MS spectral database. Further targeted isolation is necessary for a complete characterization of this compound.

**Figure 3:**
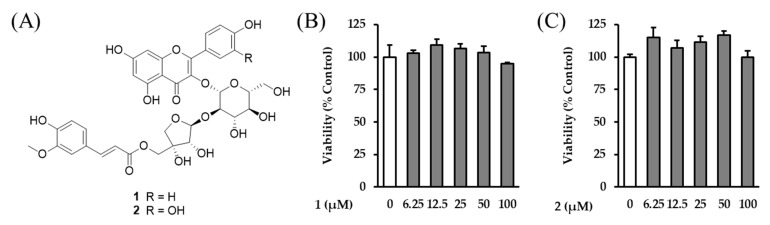
(**A**) Chemical structures of compounds **1** and **2**, isolated from the aerial parts of *A*. *membranaceus*. (**B**,**C**) NHDF cell viability in **1** (**B**) and **2** (**C**). The cells were then treated with different concentrations of the sample for 24 h. The effects of the compounds on cell viability were measured using an EZ-Cytox kit. Data are shown as mean ± SD (*n* = 3).

Using repetitive chromatography, two acylated flavonol glycosides (**1** and **2**) were isolated (Figure 3). The chemical structure of compounds **1** and **2** were identified as astraflavonoid A (**1**) and astraside C (**2**), which were first isolated from the aerial parts of *A*. *membranaceus*, comparing their spectroscopic data (HR-MS, UV, 1D- and 2D-NMR) and previously reported data [13,21]. The spectroscopic data supporting their structural identification can be found in the Supplementary Materials (Table S1).

Next, to match the major component peaks of the *A. membranaceus* aerial-part LC-MS chromatogram, we compared the isolated substances with their retention times and MS spectra. As a result, peak D was ultimately determined to be astraside C (**2**), and peak E to be astraflavonoid A (**1**).

Previous studies have demonstrated the antioxidative and anti-inflammatory effects of flavonol glycosides, which suggest their potential anti-skin-aging properties. Specifically, quercetin 3-*O*-glucoside, known as isoquercitrin, exhibits antiradical activity by scavenging ROS, which is linked to its oxidative-stress-related anti-inflammatory effects [22]. Kaempferol-3-*O*-rutinoside enhanced keratinocyte migration via FAK and Rac1 activation [23]. Kaempferol 3-*O*-glucoside inhibits collagenase, elastase, and hyaluronidase activities [24]. Additionally, rhamnocitrin 3-O-glucoside demonstrates anti-inflammatory activity against LPS-induced RAW264.7 cells [25].

Although various flavonoid glycosides have previously been reported to exert anti-skin-aging effects, astraside C (**2**) and astraflavonoid A (**1**), the major constituents isolated from the aerial parts of *A. membranaceus*, have not been studied in this context. These compounds were selected for further evaluation because they were obtained in sufficient yield and purity for biological testing, and their structural uniqueness, along with the lack of prior anti-aging data, warrants investigation of their specific pharmacological activities. In contrast, the other identified peaks (A–C, F) corresponded to known flavonoids or commercially available standards with previously reported bioactivity. Therefore, to validate the anti-aging potential of the aerial parts of *A. membranaceus*, we investigated the antioxidant and anti-inflammatory effects of compounds **1** and **2** in TNF-α-induced NHDFs.

### 2.3. Effects of Astraflavonoid A (**1**) and Astraside C (**2**) on ROS and MMP-1 Secretion in NF-α-Induced NHDF

To evaluate the anti-skin-aging properties of these two acylated flavonol glycosides—astraflavonoid A (**1**) and astraside C (**2**)—the NHDF cell viability against the compounds was investigated. It was confirmed that astraflavonoid A and astraside C (**1** and **2**) were nontoxic at concentrations of 100 μM or less (Figure 3B,C). Based on this, subsequent experiments were conducted with the astraflavonoid concentrations in this range.

**Figure 4:**
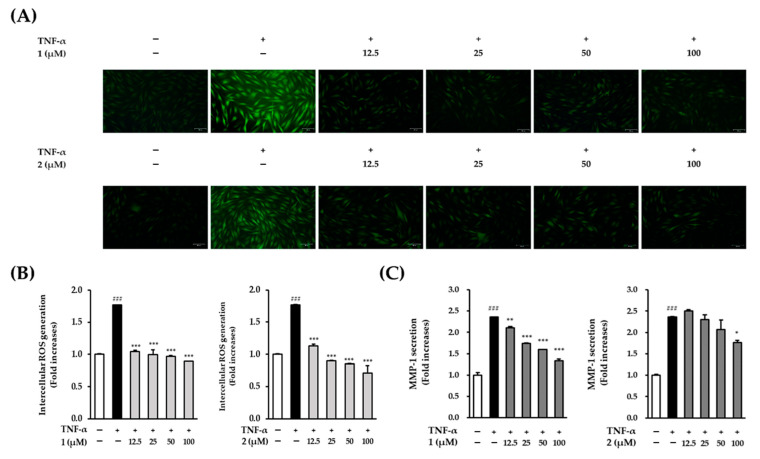
Effects of compounds **1** and **2** on ROS generation and MMP-1 secretion in TNF-α-induced NHDFs. (**A**) Fluorescence images of ROS. (**B**) Quantitative analysis of ROS levels. NHDFs were pretreated with each compound for 24 h and then stimulated with TNF-α (20 ng/mL) for 15 min. (**C**) MMP-1 secretion was measured using ELISA after 24 h of compound treatment and TNF-α stimulation. Data are presented as mean ± SEM (*n* = 2). ^###^ *p* < 0.001—untreated group versus TNF-α-induced group. * *p* < 0.05, ** *p* < 0.01, and *** *p* < 0.001—sample treatment group versus TNF-α-induced group.

Next, since the *A*. *membranaceus* extract reduced MMP-1 secretion and ROS production, the effects of astraflavonoid A (**1**) and astraside C (**2**) on TNF-α-induced ROS and MMP-1 production in NHDF were screened. Both compounds showed potential to inhibit ROS generation and reduce MMP-1 expression in TNF-α-induced NHDFs. Compared with the control group, the TNF-α treatment group showed a 1.77 ± 0.02-fold increase in the production of ROS (*p* < 0.001). The addition of astraflavonoid A (**1**) and astraside C (**2**) (100 μM) significantly reduced the amount of ROS produced, with ROS levels of 0.98 ± 0.1-fold (*p* < 0.001) and 0.71 ± 0.11-fold (*p* < 0.001), respectively, compared to the control group (Figure 4A,B), which indicates that astraflavonoid A (**1**) and astraside C (**2**) are effective against oxidative stress.

The TNF-α treatment group showed significantly increased MMP-1 secretion by 2.36 ± 0.01-fold (*p* < 0.001) compared with that of the control group. The addition of astraflavonoid A (**1**) (100 μM) significantly decreased the induced MMP-1 levels in a concentration-dependent manner [2.11 ± 0.02-fold (*p* < 0.01), 1.74 ± 0.00-fold (*p* < 0.001), 1.06 ± 0.00-fold (*p* < 0.001), and 1.34 ± 0.04-fold (*p* < 0.001) at concentrations of 12.5, 25, 50, and 100 μM, respectively], and astraside C (**2**) decreased induced MMP-1 levels to 1.76 ± 0.05-fold (*p* < 0.05) at a concentration of 100 μM (Figure 4C). Interestingly, a slight increase in MMP-1 secretion was observed at 12.5 μM for compound **2** (astraside C), which may be attributed to biological variability at lower concentrations. However, this increase was not statistically significant and did not persist at higher concentrations, confirming a clear inhibitory effect.

Thus, both compounds reduced the increase in MMP-1 secretion induced by TNF-α at a concentration of 100 μM, which indicates that astraflavonoid A (**1**) and astraside C (**2**) prevented collagen fibril decomposition by inhibiting the hypersecretion of MMP-1. In this study, MMP-1 secretion was measured using an ELISA kit that quantified the total protein level, including both active and latent forms of MMP-1. Although we did not directly assess enzymatic activity using substrate-based assays, the observed reduction in MMP-1 levels, along with decreased ROS production, indirectly suggests a potential inhibitory effect on collagen degradation. These results suggest that *A. membranaceus* extract and its major flavonoid compounds protect against TNF-α-induced oxidative stress and extracellular matrix degradation in dermal fibroblasts. Although a direct assessment of collagen degradation was not conducted in this study, the observed decrease in MMP-1 secretion suggests potential protection against collagen loss. However, as shown in Figure 3C, although both compounds exhibited concentration-dependent inhibition, astraflavonoid A (**1**) was more potent than astraside C (**2**). Compound **1** (astraflavonoid A) exhibited stronger MMP-1 inhibitory activity than compound **2**, which may be attributed to its acyl group substitutions and hydroxylation profile, which could enhance membrane permeability or binding affinity to the target enzyme. In contrast, compound **2** (astraside C) demonstrated broader anti-inflammatory effects, suggesting that these two compounds may act through different mechanisms.

### 2.4. Effects of Astraflavonoid A (**1**) and Astraside C (**2**) on IL-6 and IL-8 Secretion in TNF-α-Induced NHDFs

As we age, our levels of proinflammatory cytokines, such as IL-6 and IL-8, gradually increase [26]. These cytokines stimulate the secretion of MMP-1 and MMP-3 [27]. Thus, the effects of astraflavonoid A (**1**) and astraside C (**2**) on the amount of IL-6 and IL-8 secreted by NHDFs induced by TNF-α were investigated. The TNF-α treatment group significantly increased the secretion of IL-6 and IL-8 [9.76 ± 0.24 ng/mL (*p* < 0.001) and 3.22 ± 0.20 ng/mL (*p* < 0.001), respectively] compared with the control group.

**Figure 5:**
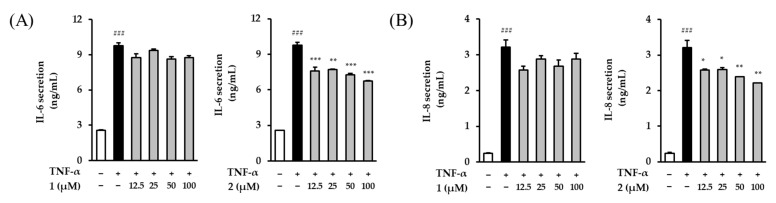
Effects of compounds **1** and **2** on IL-6 (**A**) and IL-8 (**B**) secretion in TNF-α-induced NHDFs. Cells were treated with 12.5, 25, 50, or 100 μM of each compound for 12 h. Cytokine levels were measured using ELISA. Data are presented as mean ± SEM (*n* = 2). ^###^ *p* < 0.001—untreated group versus TNF-α-induced group. * *p* < 0.05, ** *p* < 0.01, and *** *p* < 0.001—sample treatment group versus TNF-α-induced group.

In contrast to the results of the MMP-1 studies, astraflavonoid A (**1**) had no significant effect on TNF-α-induced IL-6 and IL-8 secretion, while 100 μM of astraside C (**2**) reduced the level of IL-6 and IL-8 secretion to 6.74 ± 0.06 ng/mL (*p* < 0.001) and 2.21 ± 0.00 ng/mL (*p* < 0.01), respectively (Figure 5). Astraside C (**2**) tended to decrease IL-6 and IL-8 in a concentration-dependent manner and was more potent than astraflavonoid A (**1**).

### 2.5. Expression of JNK, ERK, and p38 by Astraflavonoid A (**1**) and Astraside C (**2**) in TNF-α-induced NHDFs

In NHDFs induced by TNF-α, astraflavonoid A (**1**) tended to inhibit ROS and MMP-1 but did not affect the levels of secretion of IL-6 or IL-8. By contrast, astraside C (**2**) inhibited ROS, MMP-1, IL-6, and IL-8 production. Therefore, we conducted a mechanistic study focusing on astraside C (**2**).

**Figure 6:**
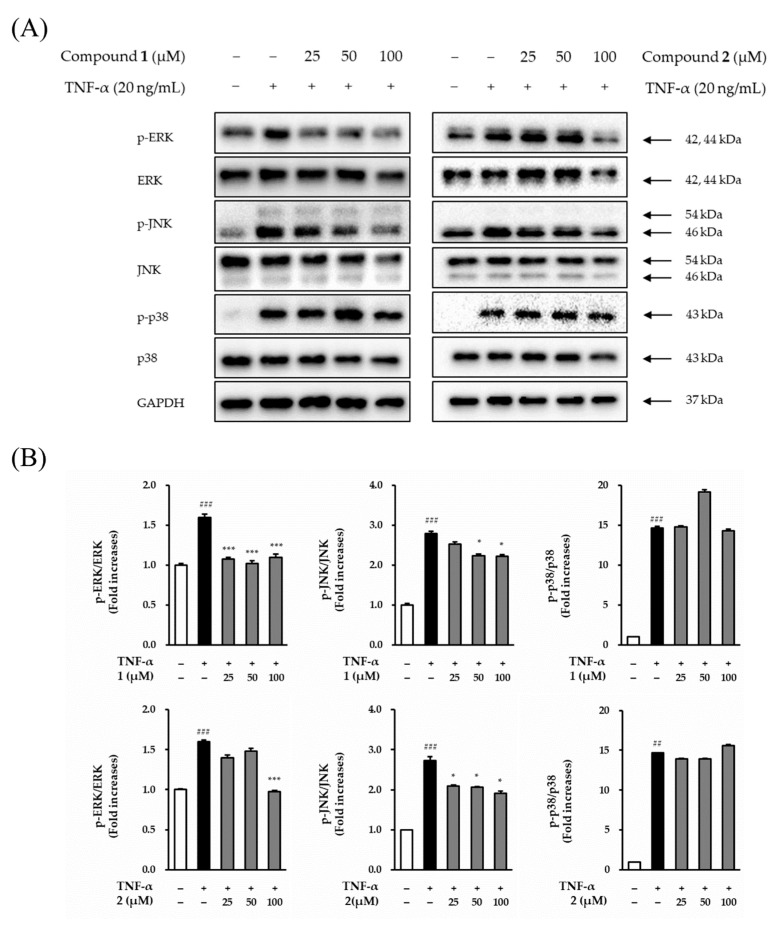
Effects of compounds **1** (left) and **2** (right) on the phosphorylation of ERK, JNK, and p38 MAPKs in TNF-α-induced NHDFs. Cells were pretreated with the compounds and then stimulated with TNF-α (20 ng/mL) for 15 min. (**A**) Western blot images. (**B**) Densitometric quantification. Data are presented as mean ± SEM (*n* = 3). ^##^ *p* < 0.01 and ^###^ *p* < 0.001—untreated group versus TNF-α-induced group. * *p* < 0.05 and *** *p* < 0.001—sample treatment group versus TNF-α-induced group.

First, the effect of astraflavonoid A (**1**) on the MAPK signaling pathway was investigated using Western blotting (Figure 6A, left). In the TNF-α treatment group, the phosphorylation of ERK increased by (1.60 ± 0.04)-fold (*p* < 0.05) compared with that in the control group. Meanwhile, although the astraflavonoid A (**1**) treatment group was not concentration-dependent, a significant suppression of ERK phosphorylation by 1.09 ± 0.04-fold (*p* < 0.001 at 100 μM) was observed. Additionally, while the phosphorylation of JNK increased by 2.73 ± 0.06-fold (*p* < 0.001) in the TNF-α treatment group compared with that in the control group, the increase was not as pronounced in the astraflavonoid A (**1**) treatment group, which showed an increase of 2.22 ± 0.04-fold (*p* < 0.05 at 100 μM). However, although p38 phosphorylation increased by 14.68 ± 0.15-fold (*p* < 0.001) in the TNF-α treatment group compared to that in the control group, there was no decrease in the astraflavonoid A (**1**) and astraside C (**2**) treatment groups.

We then investigated the effect of astraside C (**2**) on the MAPK signaling pathway using Western blotting in a similar manner (Figure 6A, right). In the TNF-α treatment group, the phosphorylation of ERK increased by 1.60 ± 0.01-fold (*p* < 0.001) compared with the control group. However, it was significantly suppressed by 0.98 ± 0.01-fold (*p* < 0.001 at 100 μM) in the astraside C (**2**) treatment group. Additionally, while the phosphorylation of JNK increased by 2.73 ± 0.09-fold (*p* < 0.01) compared with that in the control group, the increase was suppressed to 1.92 ± 0.05-fold (*p* < 0.05 at 100 μM) in the astraside C (**2**) treatment group. However, while p38 phosphorylation increased by 14.68 ± 0.01-fold (*p* < 0.01) compared with that of the control group, there was no decrease in the group treated with astraside C (**2**).

### 2.6. Expression of NF-κB, c-Jun, and COX-2 by Astraflavonoid A (**1**) and Astraside C (**2**) in TNF-α-Induced NHDFs

The MAPK signaling pathway translocates AP-1 and NF-κB, both of which increase the expression level of MMPs from the cytoplasm to the nucleus [28]. AP-1, composed of c-Fos and c-Jun or ATF (activating transcription factor) proteins, is regulated though phosphorylation in the MAPK pathway, thereby promoting the transcription of MMP genes [29]. NF-κB exists as a heterodimer of p65/p50 in dermal fibroblasts and typically bounds to its inhibitor its inhibitor, IκBα, in the cytoplasm [30]. Activated NF-κB increases the expression of MMPs and induces collagen degradation [31]. Moreover, activated NF-κB upregulates COX-2, an enzyme involved in the inflammatory process, resulting in inflammation-mediated skin disease [32]. It also participates in inflammatory responses by inducing increases in proinflammatory cytokines such as IL1, IL6, IL-8, and TNF-α [33].

**Figure 7:**
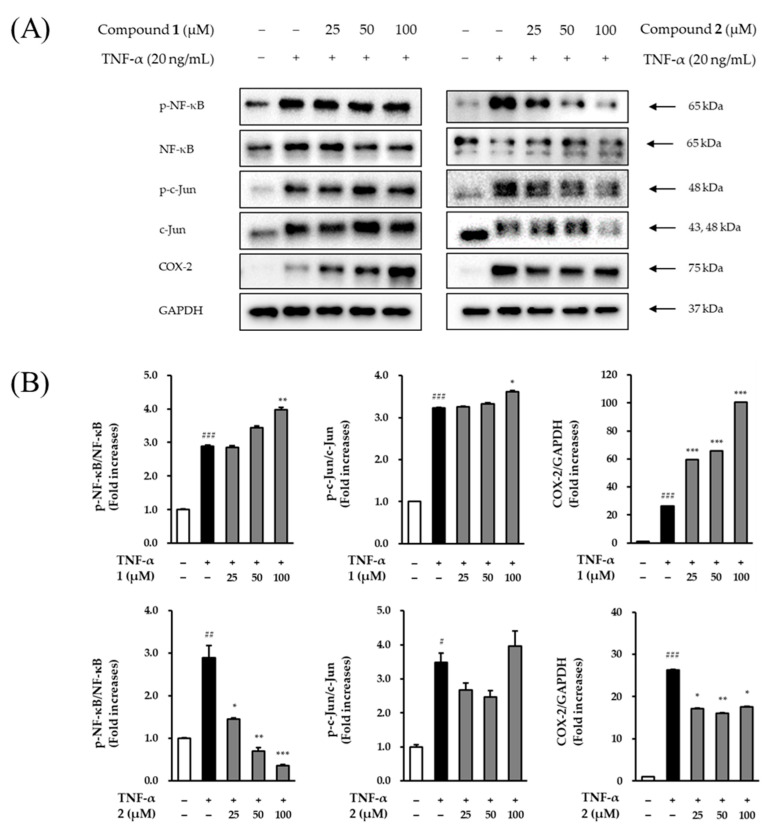
Effects of compounds **1** and **2** on expression of NF-κB, c-Jun, and COX-2 in TNF-α-induced NHDFs. Cells were treated with compounds and stimulated with TNF-α (20 ng/mL) for either 15 min (for phosphorylation) or 6 h (for total protein expression). (**A**) Western blot images. (**B**) Quantification. Data are presented as mean ± SEM (*n* = 3). ^#^ *p* < 0.05, ^##^ *p* < 0.01, and ^###^ *p* < 0.001—untreated group versus TNF-α-induced group. * *p* < 0.05, ** *p* < 0.01, and *** *p* < 0.001—sample treatment group versus TNF-α-induced group.

We initially investigated the effect of astraflavonoid A (**1**) on the NF-κB, c-Jun, and COX-2 signaling pathways using Western blotting (Figure 7A, left). In the TNF-α treatment group, the phosphorylation of NF-κB increased by 2.89 ± 0.04-fold (*p* < 0.001) compared with the control group; this was significantly increased by 3.98 ± 0.06-fold (*p* < 0.01 at 100 μM) in the astraflavonoid A (**1**) treatment group. Additionally, the phosphorylation of c-Jun increased by 3.23 ± 0.02-fold (*p* < 0.001) compared with that in the control group, whereas in the astraflavonoid A (**1**) treatment group, there was a tendency for this to increase further in a concentration-dependent manner [100 μM; 3.61 ± 0.03-fold (*p* < 0.05)]. Additionally, while the expression of COX-2 increased by 26.29 ± 0.05-fold (*p* < 0.001) compared with that in the control group, in the astraflavonoid A (**1**) treatment group this increase in expression tended to accelerate to 57.83 ± 0.05-fold (*p* < 0.001 at 25 μM), 63.82 ± 0.01-fold (*p* < 0.001 at 50 μM), and 99.83 ± 0.07-fold (*p* < 0.001 at 100 μM).

**Scheme 1:**
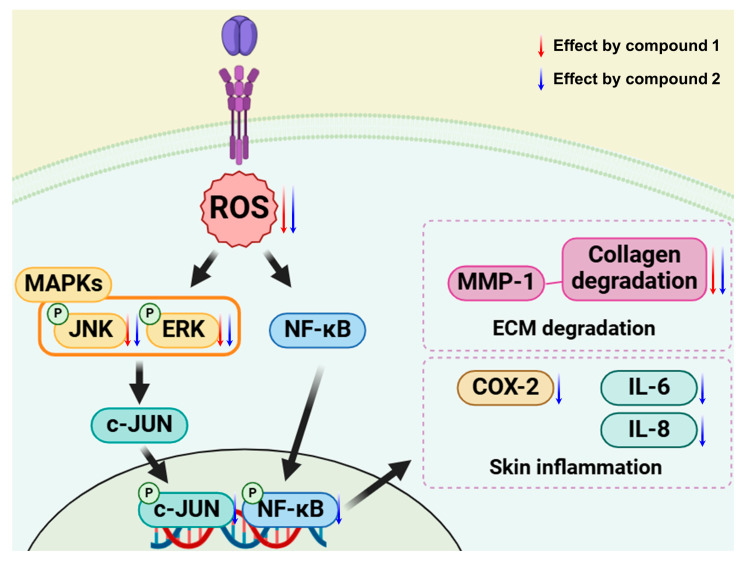
A schematic diagram of the effects of astraflavonoid (**1**) and astraside C (**2**) in TNF-α-stimulated NHDFs.

We then investigated the effect of astraside C (**2**) on the NF-κB, c-Jun, and COX-2 signaling pathways using Western blotting (Figure 7A, right). In the TNF-α treatment group, the phosphorylation of NF-κB increased by 2.89 ± 0.28-fold (*p* < 0.01) compared with that in the control group; furthermore, in the astraside C (**2**) treatment group, this tended to decrease to 1.45 ± 0.03-fold (*p* < 0.05 at 25 μM), 0.69 ± 0.09-fold (*p* < 0.01 at 50 μM), and 0.36 ± 0.03-fold (*p* < 0.001 at 100 μM). Additionally, while the phosphorylation of c-Jun increased by 3.25 ± 0.16-fold (*p* < 0.05) compared with that in the control group, in the astraside C (**2**) treatment group the increase was reduced to 2.55 ± 0.11-fold at 25 μM and 2.38 ± 0.10-fold at 50 μM. However, at 100 μM, the phosphorylation of c-Jun increased to 3.48 ± 0.26-fold that of the control. The expression of COX-2 increased by 26.29 ± 0.14-fold (*p* < 0.001) over the control group, whereas in the astraside C (**2**) treatment group, COX-2 expression was reduced to 13.25 ± 0.11-fold (*p* < 0.05 at 25 μM), 12.44 ± 0.11-fold (*p* < 0.01 at 50 μM), and 13.65 ± 0.11-fold (*p* < 0.05 at 100 μM) (Figure 7). Therefore, astraside C (**2**) appears to be effective against inflammation-related factors, providing antioxidant effects that significantly inhibit ROS production, inflammatory cytokines, MAPK, NF-κB, and COX-2. This indicates that although both compounds decrease the expression of ROS and MMP-1, downstream pathways, such as NF-κB, c-Jun, and COX-2, show opposing patterns through different mechanisms (Scheme 1).

### 2.7. Molecular Docking Study of Astraflavonoid A (**1**) and Astraside C (**2**) with COX-2

**Figure 8:**
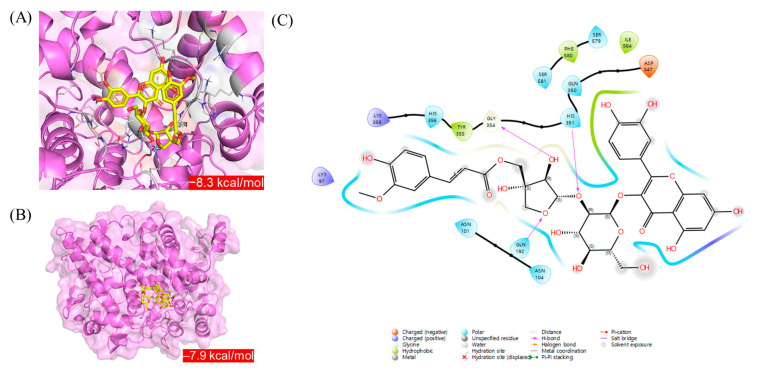
Molecular docking of compounds **2** (astraside C) and **1** (astraflavonoid A) with COX-2. (**A**,**B**) Three-dimensional binding poses of compounds **2** and **1**. Each ligand was modeled as a stick with the following colors: yellow for carbon and red for oxygen. Hydrogen bonds with GLN-192, HIS-351, and GLY-354 are indicated by cyan dotted lines. (**C**) Two-dimensional interaction diagram of astraside C–COX-2 complex, highlighting key residues involved in polar, electrostatic, and hydrophobic interactions. Two-dimensional diagrams of astraside C (**2**) and COX-2 complex at the lowest energy conformation (RMSD < 1.0). The interacting residues are labeled with the following colors: green for hydrophobic residues, blue for polar residues, orange for negatively charged residues, purple for positively charged residues, white for glycine.

In silico molecular docking simulations were conducted to compare the interactions of astraflavonoid A (**1**) and astraside C (**2**) with COX-2. Each compound bound COX-2 within the binding pocket, as shown in Figure 8. Among the best docked positions of astraflavonoid A (**1**) and astraside C (**2**), astraside C (**2**) exhibited stronger binding affinity (−8.6 kcal/mol; Figure 8A). In the astraside C (**2**)–COX-2 complex, hydrogen-bonding interactions occurred with residues GLN-192, HIS-351, and GLY-354. Polar interactions were also observed with residues ASN-101, ASN-104, GLN-192, GLN-350, HIS-351, HIS-356, SER-579, and SER-581. Additionally, the presence of charged residues (positive: LYS-97 and LYS-358; negative: ASP-347) at the potential binding site of COX-2 suggests that electrostatic interactions stabilize the complex. Hydrophobic interactions were observed for residues TYR-355, ILE-564, and PHE-580 (Figure 8C). Molecular docking analysis revealed that compound **2** (astraside C) exhibited strong binding affinity for COX-2. It formed stable hydrogen bonds with key residues, such as GLN-192, HIS-351, and GLY-354, along with additional polar, electrostatic, and hydrophobic interactions. These binding features were consistent with the COX-2 inhibitory effects observed in biological assays, supporting its anti-inflammatory potential at the molecular level.

Although this study focused on the individual effects of astraflavonoid A (**1**) and astraside C (**2**), further studies are required to evaluate their potential synergistic interactions. Future work will include co-treatment assays and quantitative approaches, such as combination index (CI) analysis, to assess additive or synergistic effects, providing a more comprehensive understanding of the pharmacological potential of *A. membranaceus* aerial parts.

## 3. Materials and Methods

### 3.1. Plant Material

The aerial parts (stems and leaves) of *A*. *membranaceus* were collected at a farm—Daedeok-myeon, Anseong-Si, Kyunggi-do, Korea (37°02′30.36″ N, 127°22′06.34″ E)—in May 2022. Prof. Dae Sik Jang authenticated the origin of the plant materials. A voucher specimen (ASME4-2022) was deposited at the College of Pharmacy, Kyung Hee University, Republic of Korea.

### 3.2. UHPLC-PDA-MS Analysis

To prepare crude extracts, 5.0 g of the aerial part of *A*. *membranaceus* was weighed and extracted using ×10 (*w*/*v*) 30% EtOH for 2 h in a sonicator. The extracts were then filtered through a filter paper and concentrated using a rotary evaporator. The final weight of the extracts was 1.29 g (yield: 25.8%). The obtained extracts were dissolved in 50% MeOH at a concentration of 10 mg/mL, filtered through a PTFE filter, and used as analytical samples. The current analysis used the Thermo Vanquish UHPLC-PDA system and LTQ-XL-iontrap-MS^n^ system, equipped with an H-ESI II probe and Thermo Hypersil GOLD column (1.9 μm, 150 mm × 2.1 mm I.D.). The injection volume of the sample was 2.0 µL. For the separation, the mobile phase consisted of solvent A (0.1% formic acid in water) and solvent B (0.1% formic acid in acetonitrile, ACN). Peak separation was achieved using a linear gradient elution with the column temperature at 40 °C: 20% B from 0 to 1.0 min, 20% to 100% B from 1.0 to 20.0 min, 100% B from 21.0 to 26.0 min, and 100% to 20% B from 26.0 to 26.5 min. The initial conditions were held for 3.5 min.

### 3.3. Extraction and Isolation of Compounds **1** and **2**

The dried aerial parts (3.0 kg) from *A*. *membranaceus* were extracted with 50% EtOH (10-fold, *w*/*v*) at room temperature for 3 h, and this was repeated three times. The mixture was filtered through No. 2 filter paper and evaporated under vacuum at 45.0 °C. The resulting extract solution was evaporated to yield a concentrated solution (ASME-A; 836.5 g, yield: 27.8%).

ASME-A was separated using Diaion HP-20 column chromatography (CC) (ϕ 10.5 × 70.0 cm, MeOH/H_2_O = 0:10–10:0), resulting in 17 fractions (F1–F17). For fraction F11, a Sephadex LH-20 CC (ϕ 4.6 × 59.0 cm, MeOH/H_2_O = 5:5–10:0) was employed, producing 14 subfractions (F11-1 to F11-14). F11-9 was further fractionated using silica MPLC (120 g, CH_2_Cl_2_/(90% MeOH) = 10:0–0:10) to yield nine subfractions (F11-9-1 to F11-9-9). F11-9-8 was subjected to RP MPLC (46 g, MeOH/H_2_O = 4:6–5:5) to isolate compounds **1** (882.1 mg, yield: 0.1055%) and **2** (45.1 mg, yield: 0.0053%).

#### 3.3.1. Astraflavonoid A (**1**)

Yellow powder; HR-ESI-MS (negative mode): *m*/*z* 755.1810 [M−H]^−^ (*m*/*z* calcd for C_36_H_35_O_18_: 755.1829); for ^1^H and ^13^C NMR data, see Table S1.

#### 3.3.2. Astraside C (**2**)

Yellow powder; HR-ESI-MS (negative mode): *m*/*z* 771.1780 [M−H]^−^ (*m*/*z* calcd for C_36_H_35_O_19_: 771.1778); UV (MeOH) λ_max_ (log *ε*): 218 (3.42), 271 (2.60), 330 (2.47) nm; for ^1^H and ^13^C NMR data, see Table S1.

### 3.4. Cell Culture

The properties of the tested extracts and compounds from the aerial parts of *A. membranaceus* were evaluated in NHDF cells purchased from PromoCell GmbH (Sickingenstr, Heidelberg, Germany). The cell lines were maintained in Dulbecco’s Modified Eagle Medium (DMEM; Corning, Manassas, VA, USA) supplemented with 10% fetal bovine serum (FBS; Atlas, Fort Collins, CO, USA) and 1% antibiotics (Pen Strep; Gibco, Grand Island, NY, USA) at 37 °C in a humidified atmosphere of 5% CO_2_.

### 3.5. Cell Viability

Cells were plated in 96-well culture plates at 1 × 10^4^ cells/well and cultured for 24 h. The medium was replaced with a serum-free medium, and the cells were incubated for 24 h. After 24 h, the cells were treated with the extract and compounds diluted in a serum-free medium. To measure cell viability, the cells were assayed by measuring 10% EZ-Cytox solution (DoGenBio, Seoul, Republic of Korea) in a serum-free medium and incubated for 1 h. The absorbance was measured at 450 nm with an EnSpire multimode plate reader (PerkinElmer, Waltham, MA, USA).

### 3.6. Intracellular ROS Generation Assay

The cells were plated in 96-well culture black plates at a density of 1 × 10^4^ cells/well and cultured for 24 h. The medium was replaced with a serum-free medium, and the cells were incubated for 24 h. After incubation, cells were pretreated with 12.5, 25, 50, and 100 μg/mL of the extract and compounds for 1 h, followed by treatment with 20 ng/mL TNF-α and 10 μM 2′,7′-dichlorodihydrofluorescein diacetate (DCFDA; Sigma-Aldrich, Burlington, NJ, USA) for 15 min at 37 °C. TNF-α stimulation was limited to 15 min in the ROS assay because of the rapid onset and short half-life of ROS generation. In contrast, later time points (6–24 h) were used for cytokines and MMPs to capture the transcriptional and translational responses associated with prolonged inflammatory signaling. Following incubation, the cells were washed once with phosphate-buffered saline (PBS), and intracellular ROS levels were measured using an EnSpire multimode plate reader (PerkinElmer, Waltham, MA, USA) at excitation and emission wavelengths of 485 and 530 nm, respectively. Fluorescence values obtained from the DCFDA assay were normalized to the TNF-α-treated group, which was defined as 100%, to ensure consistent comparisons across experimental conditions. For imaging, the cells were plated in 48-well culture plates at a density of 2 × 10^4^ cells/well and cultured for 24 h. The medium was replaced with a serum-free medium, and the cells were incubated for 24 h. The cells were pretreated with 25, 50, and 100 μg/mL (μM) of the compounds for 1 h, followed by treatment with 20 ng/mL TNF-α and 10 μM DCFDA for 15 min at 37 °C. After 15 min, the cells were washed once with phosphate-buffered saline (PBS). Images of intracellular ROS levels were obtained using a CKX53 microscope (Olympus, Tokyo, Japan).

### 3.7. Enzyme-Linked Immunosorbent Assay (ELISA)

Cells were plated in 48-well culture plates at 2 × 10^4^ cells/well and cultured for 24 h. The medium was replaced with serum-free medium, and the cells were incubated for 24 h. After 24 h, cells were pretreated with 12.5, 25, 50, and 100 μg/mL of extract and compounds for 1 h, followed by treatment with 20 ng/mL TNF-α for 12 and 24 h incubation at 37 °C. Following incubation, the cell culture medium from each well was analyzed using MMP-1, pro-collagen type I α1, IL-6, and IL-8 ELISA assay kits (R&D Systems, Inc., Minneapolis, MN, USA). The experiments were performed according to the manufacturer’s instructions. Absorbance was measured at 450 nm using an EnSpire multimode plate reader (PerkinElmer, Waltham, MA, USA).

### 3.8. Western Blotting

The cells were plated in 6-well culture plates at 3 × 10^5^ cells/well and cultured for 24 h. The medium was replaced with a serum-free medium, and the cells were incubated for 24 h. After 24 h, the cells were pretreated with 25, 50, and 100 μM of compounds **1** and **2**, respectively, for 1 h, followed by treatment with 20 ng/mL TNF-α for 15 min and 6 h of incubation at 37 °C. Following incubation, the cells were lysed in lysis buffer (RIPA; Tech & Innovation, Gangwon, Republic of Korea), and the lysate protein concentrations were quantified using a BCA protein assay kit (Thermo Scientific, Waltham, MA, USA). Proteins were separated by sodium dodecyl sulfate–polyacrylamide gel electrophoresis (SDS-PAGE) and transferred to polyvinylidene fluoride (PVDF) membranes. Membranes were blocked with 5% skimmed milk in Tris-buffered saline-Tween 20 (TBS-T) for 1 h, washed with TBS-T, and incubated with primary antibodies overnight at 4 °C. The membrane was then incubated with the secondary antibody at room temperature for 2 h. Proteins on the membrane were detected with a Clarity Western ECL Substrate (Hercules, CA, USA). The following primary antibodies were used: phospho-ERK, ERK, phospho-JNK, JNK, phospho-p38, p38, phospho-NF-κB, NF-κB, phospho-c-Jun, c-Jun, COX-2, and GAPDH (Cell Signaling Technology, Danvers, MA, USA).

### 3.9. Statistical Analysis

Statistical analyses were performed using GraphPad Prism version 8.0.1 (GraphPad Software Inc., La Jolla, CA, USA). Data are presented as mean ± standard deviation (SD) or standard error of the mean (SEM). Data were analyzed using one-way analysis of variance (ANOVA), and statistical significance was defined using Tukey’s test at *p* < 0.05.

### 3.10. Molecular Docking

Molecular docking studies of compounds **1** and **2** with COX-2 (PDB ID: 5IKR) were conducted using AutoDock Vina (ver 1.1.2, The Scripps Research Institute, La Jolla, CA, USA) following a previously described method [34]. Briefly, each ligand, compounds **1** and **2**, and the co-crystallized ligand were prepared by MMFF994 force field minimization. COX-2 was prepared by removing the water molecules and adding polar hydrogen atoms. Next, a molecular docking simulation was performed using the hybrid Lamarckian Genetic Algorithm (LGA). After the docking simulation, 3D and 2D diagrams of the COX-2-ligand complex were generated using PyMOL (ver 2.5, Schrödinger, LLC, New York, NY, USA) and Maestro software (ver 12.9, Schrödinger).

## 4. Conclusions

In conclusion, the present study demonstrates the potential of utilizing the aerial parts of *A*. *membranaceus* as therapeutic agents for anti-aging and anti-inflammatory skin treatment. The 30% ethanol extract obtained from these aerial parts notably reduced ROS and MMP-1 expression in NHDF cells. Chemical profiling identified flavonol glycosides as the major constituents of the aerial parts of *A*. *membranaceus* extract, with the isolation of astraflavonoid A (**1**) and astraside C (**2**), which were isolated for the first time from the aerial part. Astraflavonoid A (**1**) and astraside C (**2**), the anti-skin-aging and anti-inflammatory properties of which were previously unknown, inhibit ROS and MMP-1. Astraside C (**2**) significantly reduced the expression of IL-6 and IL-8, indicating its strong anti-inflammatory potential. These findings suggest that astraside C (**2**) may serve as a promising candidate for anti-skin-aging and anti-inflammatory therapies by modulating key pathways, including MAPK, NF-κB, and COX-2, thereby targeting both intrinsic and extrinsic aging factors (Scheme 1). Although both compounds reduced intracellular ROS levels, their underlying anti-inflammatory mechanisms appear to differ. Compound **1** primarily inhibited ERK and JNK phosphorylation, while compound **2** more effectively suppressed NF-κB, c-Jun, and COX-2, which is likely attributed to structural differences—particularly the additional hydroxyl group on the B-ring of compound **2**.

This study was conducted using NHDFs, which limits the interpretation of the upstream and downstream signaling mechanisms. Moreover, since the skin consists of both the dermis and epidermis, additional studies on keratinocytes and melanocytes, which play essential roles in the epidermis, are necessary to fully understand skin-aging-related responses. To further confirm the physiological relevance and translational potential of our findings, future research should include in vivo animal studies and the application of 3D reconstructed human skin models composed of multiple skin cell types. These approaches provide a more comprehensive understanding of the biological effects and underlying mechanisms in a physiologically relevant environment.

## Figures and Tables

**Table 1:** Chemical profiling of the major constituents in the 30% EtOH extract from aerial parts of *A*. *membranaceus* (ASME-A).

Peak	Identification	*t*_R_ (min)	UV (λ_max_, nm)	Chemical Formula	Observed *m*/*z* [M + H]^+^	MS/MS Fragment Ions
A	Quercetin-3-*O*-glucoside	11.85	206, 258, 354	C_21_H_20_O_12_	465.0	303
B	Kaempferol-3-*O*-rutinoside	12.35	206, 266, 354	C_27_H_30_O_15_	595.0	287, 448
C	Kaempferol-3-*O*-glucoside	14.38	206, 266, 354	C_21_H_20_O_11_	449.1	287
D	Astraside C (**2**)	17.85	206, 254, 266, 334	C_36_H_36_O_19_	773.0	291, 303, 507, 755
E	Astraflavonoid A (**1**)	19.72	206, 270, 326	C_36_H_36_O_18_	757.0	287, 291, 449, 595, 637, 739
F	Rhamnocitrin-3-*O*-glucoside	24.58	206, 266, 354	C_22_H_22_O_11_	463.0	301

## Data Availability

Data are available upon request.

## Supplementary Materials

The following supporting information can be downloaded at: https://www.mdpi.com/article/10.3390/plants14091358/s1: Figure S1: Chemical structure of putatively annotated peaks A, B, C, and F in ASME-A by LC-MS/MS data; Table S1: ^1^H and ^13^C NMR spectroscopic data of compounds **1** and **2** (*δ* in ppm, methanol-*d*_4_, 500 and 125 MHz).
