# Supplementary material for: Effects of Astraflavonoid A and Astraside C from the Aerial Part of Astragalus membranaceus on TNF-α-Induced Human Dermal Fibroblasts
Source: Plants (Basel). 2025 Apr 30;14(9):1358. doi: 10.3390/plants14091358 (PMC12074202; doi:10.3390/plants14091358)
Supplement: Supplementary file 1 [file plants-14-01358-s001.zip › plants-3574215-supplementary.pdf]

Supplementary Materials for

Effects of Astraflavonoid A and Astraside C from the Aerial Part of *Astragalus membranaceus* on TNF-α-Induced Human Dermal Fibroblasts

So-Ri Son [1], Kang Sub Kim [2], Mingoo Jun [3], Dae Sik Jang [1,3,*] and Sullim Lee [4,*]

[1] College of Pharmacy, Kyung Hee University, 26 Kyungheedae-ro, Dongdaemun-gu, Seoul 02453, Republic of Korea; allosori@khu.ac.kr
[2] College of Korean Medicine, Gachon University, 1342 Seongnamdae-ro, Sujeong-gu, Seongnam 13120, Republic of Korea; kang-sub@gachon.ac.kr
[3] Department of Biomedical and Pharmaceutical Sciences, Graduate School, Kyung Hee University, 26 Kyungheedae-ro, Dongdaemun-gu, Seoul 02453, Republic of Korea; mingoojun0104@khu.ac.kr
[4] Department of Life Science, College of Bio-Nano Technology, Gachon University, 1342 Seongnamdae-ro, Sujeong-gu, Seongnam 13120, Republic of Korea
* Correspondence: dsjang@khu.ac.kr (D.S.J.); sullimlee@gachon.ac.kr (S.L.)

Table S1. ^{1}H- and ^{13}C-NMR spectroscopic data of compounds **1** and **2** (δ in ppm, methanol-d_4, 500 and 125 MHz).

**Position**	**1**		**2**	
	δ_H Multi (J in Hz)	δ_C	δ_H Multi (J in Hz)	δ_C
2		158.0		157.7
3		134.7		134.6
4		179.3		179.0
5		163.3		163.0
6	6.08 d (2.0)	100.5	6.00 d (2.0)	99.5
7		165.5		165.3
8	6.10 d (2.0)	94.7	6.03 d (2.0)	94.4
9		158.3		157.9
10		106.3		106.0
1′		123.3		123.4
2′	8.00 d (8.5)	132.3	7.59 d (2.0)	117.2
3′	6.97 d (8.5)	116.2		145.9
4′		161.5		149.4
5′	6.97 d (8.5)	116.2	6.81 d (8.0)	115.9
6′	8.00 d (8.5)	132.3	7.53 dd (8.0, 2.0)	123.2
**3-*O*-Glc**				
1″	5.75 d (7.5)	99.8	5.70 d (8.0)	100.4
2″	3.70 m overlapped	77.4	3.70 m overlapped	77.1
3″	3.59 t (9.0)	78.7	3.59 t (9.0)	78.3
4″	3.29 m overlapped	72.0	3.27 m overlapped	71.6
5″	3.22 m overlapped	79.0	3.22 m overlapped	78.7
6″	3.72 m overlapped<br>3.51 dd (12.0, 6.0)	62.8	3.72 m overlapped<br>3.52 dd (12.0, 6.0)	62.5
**2″-*O*-Api**				
1‴	5.50 br s	109.6	5.48 s	109.4
2‴	3.88 s	78.4	3.87 s	78.2
3‴		79.7		79.5
4‴	4.31 d (11.0)<br>3.69 d (11.0) overlapped	75.5	4.29 d (11.0) overlapped<br>3.70 d overlapped	75.3
5‴	4.49 d (11.0)<br>4.30 d (11.0)	70.1	4.45 d (11.0)<br>4.29 d (11.0) overlapped	70.0
**5‴-*O*-Feruloyl**				
1⁗		127.7		127.4
2⁗	6.91 d (1.5)	111.6	6.96 d (2.0)	111.3
3⁗		149.4		149.0
4⁗		150.7		150.3
5⁗	6.76 d (8.0)	116.5	6.71 d (8.0)	116.2
6⁗	6.93 dd (8.0, 1.0)	124.3	6.78 dd (8.0, 2.0)	124.1
7⁗	7.24 d (15.5)	146.9	7.18 d (16.0)	146.6
8⁗	6.05 d (15.5)	114.9	6.00 d (16.0)	114.6
9⁗		169.1		169.9
3⁗-OCH_3	3.85 s	56.5	3.80 s	56.3

Figure S1. Chemical structure of putatively annotated peaks A, B, C and F in ASME-A by LC-MS/MS data.

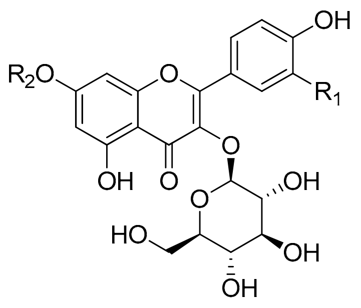

A R_1 = OH, R_2 = H
C R_1, R_2 = H
F R_1 = H, R_2 = CH_3

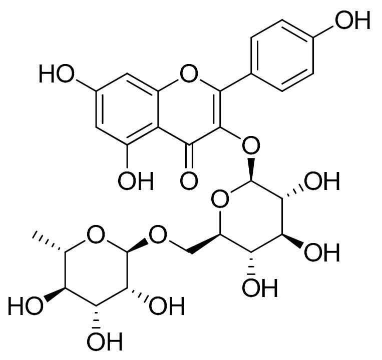

B
